# Tetra­chlorido­(1,10-phenanthroline-κ^2^ *N*,*N*′)tin(IV) 1,2-dichloro­ethane hemisolvate

**DOI:** 10.1107/S1600536809017346

**Published:** 2009-05-29

**Authors:** Badri Z. Momeni, Frank Rominger, Simin S. Hosseini

**Affiliations:** a Department of Chemistry, K. N. Toosi University of Technology, PO Box 16315-1618, Tehran 15418, Iran; b Organisch-Chemisches Institut, Universität Heidelberg, Im Neuenheimer Feld 270, 69120 Heidelberg, Germany

## Abstract

The asymmetric unit of the title compound, [SnCl_4_(C_12_H_8_N_2_)]·0.5C_2_H_4_Cl_2_, contains a tin complex and one disordered half-mol­ecule of the solvent dichloro­ethane [occupancies 0.71 (2):0.29 (2)]. The six coordinate Sn(IV) atom adopts a distorted octa­hedral geometry. π–π inter­actions between adjacent aromatic rings [interplanar distance 3.483 (5) Å] seem to be effective in the stabilization of the crystal packing.

## Related literature

For tin(IV) halide complexes with a variety of Lewis bases, see: Harrison *et al.* (1972 ▶). For 1:1 complexes of the type [Sn*X*_4_(*NN*)] (*X* = halide; *NN* = 1,10-phenanthroline or 2,2′-bipyridyl ligand), see: Matsubayashi & Iyoda (1977 ▶). For the structure of the title complex without the co-crystallized solvent, see: Su *et al.* (2007 ▶) and with co-crystallized benzene, see: Hall & Tiekink (1996 ▶). For the preparation of *trans*-[PtClMe_2_(CH_2_Cl)(phen)] used in the synthesis, see: Monaghan & Puddephatt (1985 ▶).
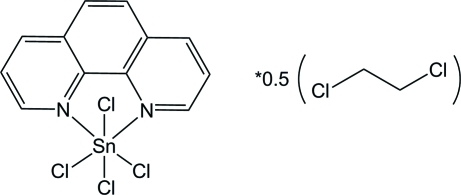


## Experimental

### 

#### Crystal data

[SnCl_4_(C_12_H_8_N_2_)]·0.5C_2_H_4_Cl_2_
*M* *_r_* = 490.17
Orthorhombic, 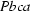

*a* = 14.4478 (2) Å
*b* = 12.3681 (1) Å
*c* = 18.3551 (2) Å
*V* = 3279.91 (6) Å^3^
*Z* = 8
Mo *K*α radiation
μ = 2.37 mm^−1^
*T* = 200 K
0.20 × 0.18 × 0.12 mm

#### Data collection

Bruker SMART CCD diffractometer
Absorption correction: multi-scan (*SADABS*; Sheldrick, 2008*a* ▶) *T*_min_ = 0.649, *T*_max_ = 0.765
31457 measured reflections
3747 independent reflections
2954 reflections with *I* > 2σ(*I*)
*R*_int_ = 0.058

#### Refinement

*R*[*F* ^2^ > 2σ(*F* ^2^)] = 0.032
*wR*(*F* ^2^) = 0.085
*S* = 1.05
3747 reflections
195 parameters
H-atom parameters constrained
Δρ_max_ = 0.43 e Å^−3^
Δρ_min_ = −1.16 e Å^−3^

### 

Data collection: *SMART* (Bruker, 1995 ▶); cell refinement: *SAINT* (Bruker, 1995 ▶); data reduction: *SAINT*; program(s) used to solve structure: *SHELXTL* (Sheldrick, 2008*b* ▶); program(s) used to refine structure: *SHELXTL*; molecular graphics: *SHELXTL*; software used to prepare material for publication: *SHELXTL*.

## Supplementary Material

Crystal structure: contains datablocks I, global. DOI: 10.1107/S1600536809017346/hg2511sup1.cif

Structure factors: contains datablocks I. DOI: 10.1107/S1600536809017346/hg2511Isup2.hkl

Additional supplementary materials:  crystallographic information; 3D view; checkCIF report

## Figures and Tables

**Table 1:** Selected geometric parameters (Å, °)

Sn1—N21	2.224 (3)
Sn1—N11	2.238 (3)
Sn1—Cl2	2.3333 (12)
Sn1—Cl1	2.3708 (10)
Sn1—Cl4	2.4095 (10)
Sn1—Cl3	2.4480 (10)

**Fig. 1.**
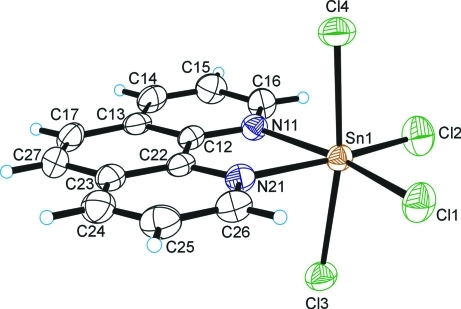
The disordered solvent molecule is omitted for clarity. Displacement ellipsoids are drawn at 50% probability level.

**Fig. 2.**
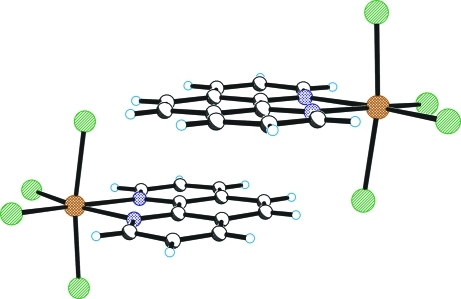
A pair of two molecules in the crystal packing showing π-π interactions. The (symmetry imposed) parallel and overlapping phenanthroline planes have a distance of 3.483 (5) Å.

## supplementary crystallographic information

### Comment

It has long been known that tin(IV) halides form complexes with a variety of Lewis bases (Harrison *et al.*, 1972). Bidentate diimine ligandes are one of the strongest bases towards tin(IV) halides and more often form 1:1 complexes of the type [Sn*X*_4_(NN)] (*X* = halide; NN = diimine ligand). Among them, the 1,10-phenanthroline and 2,2'-bipyridyl ligands are of particular interest (Matsubayashi & Iyoda, 1977). The title compound reported here, an adventitious result of our work on organoplatinum complexes, has a distorted octahedral geometry including different Sn—Cl and Sn—N bond lengths (see Fig. 1). The Cl3—Sn—Cl4 geometry shows remarkable deviation from linearity with a bond angle of 170.32 (4)°. The contraction of N11—Sn1—N21 to 74.7 (1) from the ideal 90° is typical for a chelating phenanthroline ligand. Figure 2 depicts the π-π interaction between adjacent aromatic rings, which seems, among Cl···Cl van der Waals contacts, to be significant in the stabilization of the crystal packing, as both preliminary structure determinations of the same complex, either without cocrystallized solvent (Su *et al.*, 2007) or with co-crystallized benzene (Hall & Tiekink, 1996) show the same intermolecular contact features.

### Experimental

A solution of SnCl_2_*2H_2_O (40 mg, 0.18 mmol) in THF (1 ml) and a solution of PPh_3_ (26 mg, 0.10 mmol) in dichloromethane (1 ml) were added to a dichloromethane solution (10 ml) of *cis*- and *trans*-[PtClMe_2_{CH_2_Cl}(phen)] (50 mg, 0.10 mmol) (Monaghan & Puddephatt, 1985) under Argon atmosphere. The reaction mixture was stirred for 3 h whereupon the yellow solution turned colourless. The solvent was removed under vacuum and the resulting white oily residue was solidified from CH_2_Cl_2_-diethylether solution to afford *trans*-[PtMe_2_(CH_2_Cl)(phen)(PPh_3_)][SnCl_3_]* C_2_Cl_2_H_4_. Yield: 85%; m.p. 429 K.  Anal. Calc. for C_35_H_35_Cl_6_N_2_PPtSn: C, 40.4; H, 3.4; N, 2.7. Found: C, 39.7; H, 3.0; N, 2.5. NMR data in 1,2-dichloroethane/CDCl_3_: δ (^31^P) 1.50 [^1^J(^195^Pt-^31^P) = 1036 Hz]. The title complex crystallized during the slow decomposition of the organoplatinum(IV) species from a 1,2-dichloroethane solution yielding yellow polyhedral crystals.

### Refinement

For all hydrogen atoms the positions were calculated according to geometrical criteria. During the refinement the hydrogen atoms were allowed to shift with the parent C atoms with C-H - 0.95-0.98Å. The isotropic displacement parameters were set as 1.2 times the equivalent isotropic displacement parameters of the parent atoms.

The solvent molecule 1,2-dichloroethane was found to be situated on an centre of inversion. In the final structure model the ethylene unit of the solvent molecule shows disorder over two different conformations with occupancies of 71 (2)% and 29 (2)%, respectively.

### Crystal data

**Table:**

[SnCl_4_(C_12_H_8_N_2_)]·0.5C_2_H_4_Cl_2_	*D*_x_ = 1.985 Mg m^−^^3^
*M**_r_* = 490.17	Melting point: 429 K
Orthorhombic, *P**b**c**a*	Mo *K*α radiation, λ = 0.71073 Å
Hall symbol: -P 2ac 2ab	Cell parameters from 7233 reflections
*a* = 14.4478 (2) Å	θ = 3.3–24.3°
*b* = 12.3681 (1) Å	µ = 2.37 mm^−^^1^
*c* = 18.3551 (2) Å	*T* = 200 K
*V* = 3279.91 (6) Å^3^	Polyhedron, yellow
*Z* = 8	0.20 × 0.18 × 0.12 mm
*F*(000) = 1896	

### Data collection

**Table:**

Bruker SMART CCD diffractometer	3747 independent reflections
Radiation source: fine-focus sealed tube	2954 reflections with *I* > 2σ(*I*)
graphite	*R*_int_ = 0.058
ω scans	θ_max_ = 27.5°, θ_min_ = 2.2°
Absorption correction: multi-scan (*SADABS*; Sheldrick, 2008a)	*h* = −18→18
*T*_min_ = 0.649, *T*_max_ = 0.765	*k* = −16→16
31457 measured reflections	*l* = −23→23

### Refinement

**Table:**

Refinement on *F*^2^	Primary atom site location: structure-invariant direct methods
Least-squares matrix: full	Secondary atom site location: difference Fourier map
*R*[*F*^2^ > 2σ(*F*^2^)] = 0.032	Hydrogen site location: inferred from neighbouring sites
*wR*(*F*^2^) = 0.085	H-atom parameters constrained
*S* = 1.05	*w* = 1/[σ^2^(*F*_o_^2^) + (0.0328*P*)^2^ + 8.0328*P*] where *P* = (*F*_o_^2^ + 2*F*_c_^2^)/3
3747 reflections	(Δ/σ)_max_ = 0.001
195 parameters	Δρ_max_ = 0.43 e Å^−^^3^
0 restraints	Δρ_min_ = −1.16 e Å^−^^3^

### Special details

**Table:**

Geometry. All e.s.d.'s (except the e.s.d. in the dihedral angle between two l.s. planes) are estimated using the full covariance matrix. The cell e.s.d.'s are taken into account individually in the estimation of e.s.d.'s in distances, angles and torsion angles; correlations between e.s.d.'s in cell parameters are only used when they are defined by crystal symmetry. An approximate (isotropic) treatment of cell e.s.d.'s is used for estimating e.s.d.'s involving l.s. planes.
Refinement. Refinement of *F*^2^ against ALL reflections. The weighted *R*-factor *wR* and goodness of fit *S* are based on *F*^2^, conventional *R*-factors *R* are based on *F*, with *F* set to zero for negative *F*^2^. The threshold expression of *F*^2^ > σ(*F*^2^) is used only for calculating *R*-factors(gt) *etc*. and is not relevant to the choice of reflections for refinement. *R*-factors based on *F*^2^ are statistically about twice as large as those based on *F*, and *R*- factors based on ALL data will be even larger.

### Fractional atomic coordinates and isotropic or equivalent isotropic displacement parameters (Å^2^)

**Table:**

	*x*	*y*	*z*	*U*_iso_*/*U*_eq_	Occ. (<1)
Sn1	0.373296 (17)	0.288908 (19)	0.309792 (13)	0.02635 (9)	
Cl1	0.36021 (8)	0.11063 (8)	0.26347 (6)	0.0421 (3)	
Cl2	0.22059 (8)	0.34733 (10)	0.29708 (7)	0.0511 (3)	
Cl3	0.34492 (8)	0.22870 (8)	0.43486 (5)	0.0390 (2)	
Cl4	0.42343 (8)	0.36387 (9)	0.19523 (6)	0.0435 (3)	
N11	0.4206 (2)	0.4450 (2)	0.35899 (17)	0.0283 (7)	
C12	0.5099 (3)	0.4445 (3)	0.38213 (19)	0.0269 (8)	
C13	0.5508 (3)	0.5344 (3)	0.4160 (2)	0.0306 (8)	
C14	0.4951 (3)	0.6273 (3)	0.4237 (2)	0.0374 (9)	
H14	0.5197	0.6900	0.4463	0.045*	
C15	0.4064 (3)	0.6278 (3)	0.3989 (2)	0.0408 (10)	
H15	0.3695	0.6911	0.4029	0.049*	
C16	0.3698 (3)	0.5334 (3)	0.3672 (2)	0.0366 (9)	
H16	0.3073	0.5330	0.3512	0.044*	
C17	0.6451 (3)	0.5263 (3)	0.4401 (2)	0.0387 (10)	
H17	0.6726	0.5861	0.4643	0.046*	
N21	0.5220 (2)	0.2640 (2)	0.33599 (17)	0.0283 (7)	
C22	0.5637 (3)	0.3483 (3)	0.37021 (19)	0.0274 (8)	
C23	0.6563 (3)	0.3443 (3)	0.3932 (2)	0.0327 (9)	
C24	0.7061 (3)	0.2487 (4)	0.3787 (2)	0.0407 (10)	
H24	0.7690	0.2426	0.3931	0.049*	
C25	0.6635 (3)	0.1650 (4)	0.3438 (2)	0.0404 (10)	
H25	0.6967	0.1004	0.3337	0.049*	
C26	0.5708 (3)	0.1745 (3)	0.3230 (2)	0.0342 (9)	
H26	0.5418	0.1156	0.2990	0.041*	
C27	0.6951 (3)	0.4365 (4)	0.4294 (2)	0.0404 (10)	
H27	0.7574	0.4339	0.4460	0.048*	
Cl11	0.61683 (10)	0.09903 (12)	0.52665 (8)	0.0666 (4)	
C31	0.5152 (6)	0.0229 (6)	0.5363 (4)	0.049 (3)	0.71 (2)
H31A	0.5260	−0.0370	0.5711	0.058*	0.71 (2)
H31B	0.4654	0.0693	0.5563	0.058*	0.71 (2)
C31B	0.5342 (18)	0.024 (2)	0.4789 (13)	0.066 (8)*	0.29 (2)
H31C	0.5043	0.0714	0.4427	0.079*	0.29 (2)
H31D	0.5666	−0.0343	0.4517	0.079*	0.29 (2)

### Atomic displacement parameters (Å^2^)

**Table:**

	*U*^11^	*U*^22^	*U*^33^	*U*^12^	*U*^13^	*U*^23^
Sn1	0.03044 (14)	0.02244 (13)	0.02617 (14)	−0.00135 (10)	0.00109 (10)	−0.00118 (10)
Cl1	0.0475 (6)	0.0298 (5)	0.0491 (6)	−0.0029 (4)	−0.0022 (5)	−0.0091 (4)
Cl2	0.0451 (6)	0.0513 (7)	0.0567 (7)	0.0050 (5)	−0.0043 (5)	−0.0082 (6)
Cl3	0.0451 (5)	0.0396 (6)	0.0323 (5)	−0.0064 (4)	0.0046 (4)	0.0031 (4)
Cl4	0.0548 (7)	0.0421 (6)	0.0335 (5)	−0.0053 (5)	0.0050 (5)	0.0067 (5)
N11	0.0316 (17)	0.0237 (15)	0.0296 (16)	−0.0005 (13)	−0.0005 (13)	0.0005 (13)
C12	0.0291 (19)	0.0265 (18)	0.0251 (18)	−0.0028 (15)	0.0044 (15)	0.0024 (15)
C13	0.040 (2)	0.0279 (19)	0.0237 (18)	−0.0065 (16)	0.0035 (16)	0.0045 (15)
C14	0.050 (3)	0.026 (2)	0.037 (2)	−0.0082 (18)	0.0038 (19)	−0.0034 (17)
C15	0.053 (3)	0.027 (2)	0.043 (2)	0.0033 (19)	0.000 (2)	−0.0039 (18)
C16	0.038 (2)	0.030 (2)	0.042 (2)	0.0034 (17)	−0.0026 (18)	−0.0033 (17)
C17	0.042 (2)	0.038 (2)	0.036 (2)	−0.0172 (18)	−0.0013 (18)	0.0018 (18)
N21	0.0305 (17)	0.0263 (16)	0.0282 (16)	0.0008 (13)	0.0025 (13)	0.0005 (13)
C22	0.0311 (19)	0.0263 (19)	0.0246 (18)	−0.0041 (15)	0.0046 (15)	0.0046 (15)
C23	0.0290 (19)	0.038 (2)	0.032 (2)	−0.0010 (17)	0.0037 (16)	0.0092 (17)
C24	0.031 (2)	0.044 (2)	0.048 (3)	0.0015 (19)	0.0030 (19)	0.013 (2)
C25	0.039 (2)	0.035 (2)	0.047 (3)	0.0108 (19)	0.009 (2)	0.0086 (19)
C26	0.038 (2)	0.0292 (19)	0.035 (2)	0.0025 (17)	0.0072 (17)	0.0012 (16)
C27	0.032 (2)	0.047 (3)	0.041 (2)	−0.0098 (19)	−0.0041 (18)	0.009 (2)
Cl11	0.0691 (9)	0.0617 (8)	0.0691 (9)	−0.0178 (7)	0.0144 (7)	−0.0220 (7)
C31	0.061 (5)	0.048 (4)	0.037 (4)	−0.005 (3)	0.021 (3)	−0.008 (3)

### Geometric parameters (Å, °)

**Table:**

Sn1—N21	2.224 (3)	N21—C26	1.334 (5)
Sn1—N11	2.238 (3)	N21—C22	1.357 (5)
Sn1—Cl2	2.3333 (12)	C22—C23	1.404 (5)
Sn1—Cl1	2.3708 (10)	C23—C24	1.410 (6)
Sn1—Cl4	2.4095 (10)	C23—C27	1.434 (6)
Sn1—Cl3	2.4480 (10)	C24—C25	1.363 (6)
Cl1—Cl2^i^	3.5140 (16)	C24—H24	0.9500
N11—C16	1.325 (5)	C25—C26	1.397 (6)
N11—C12	1.358 (5)	C25—H25	0.9500
C12—C13	1.404 (5)	C26—H26	0.9500
C12—C22	1.438 (5)	C27—H27	0.9500
C13—C14	1.409 (6)	Cl11—C31B	1.75 (3)
C13—C17	1.436 (6)	Cl11—C31	1.754 (8)
C14—C15	1.360 (6)	C31—C31^ii^	1.513 (17)
C14—H14	0.9500	C31—H31A	0.9900
C15—C16	1.407 (6)	C31—H31B	0.9900
C15—H15	0.9500	C31B—C31B^ii^	1.38 (5)
C16—H16	0.9500	C31B—H31C	0.9900
C17—C27	1.339 (6)	C31B—H31D	0.9900
C17—H17	0.9500		
			
N21—Sn1—N11	74.74 (11)	C15—C16—H16	119.2
N21—Sn1—Cl2	168.06 (9)	C27—C17—C13	121.7 (4)
N11—Sn1—Cl2	93.56 (9)	C27—C17—H17	119.2
N21—Sn1—Cl1	91.48 (8)	C13—C17—H17	119.2
N11—Sn1—Cl1	166.22 (8)	C26—N21—C22	119.1 (3)
Cl2—Sn1—Cl1	100.18 (4)	C26—N21—Sn1	126.0 (3)
N21—Sn1—Cl4	87.22 (9)	C22—N21—Sn1	115.0 (2)
N11—Sn1—Cl4	85.89 (8)	N21—C22—C23	122.3 (4)
Cl2—Sn1—Cl4	94.46 (4)	N21—C22—C12	117.8 (3)
Cl1—Sn1—Cl4	93.95 (4)	C23—C22—C12	119.8 (4)
N21—Sn1—Cl3	85.24 (8)	C22—C23—C24	117.3 (4)
N11—Sn1—Cl3	86.29 (8)	C22—C23—C27	119.0 (4)
Cl2—Sn1—Cl3	91.70 (4)	C24—C23—C27	123.8 (4)
Cl1—Sn1—Cl3	92.29 (4)	C25—C24—C23	119.7 (4)
Cl4—Sn1—Cl3	170.32 (4)	C25—C24—H24	120.1
C16—N11—C12	119.6 (3)	C23—C24—H24	120.1
C16—N11—Sn1	126.0 (3)	C24—C25—C26	119.8 (4)
C12—N11—Sn1	114.4 (2)	C24—C25—H25	120.1
N11—C12—C13	122.3 (3)	C26—C25—H25	120.1
N11—C12—C22	118.0 (3)	N21—C26—C25	121.9 (4)
C13—C12—C22	119.7 (3)	N21—C26—H26	119.1
C12—C13—C14	116.7 (4)	C25—C26—H26	119.1
C12—C13—C17	118.7 (4)	C17—C27—C23	121.1 (4)
C14—C13—C17	124.6 (4)	C17—C27—H27	119.5
C15—C14—C13	120.6 (4)	C23—C27—H27	119.5
C15—C14—H14	119.7	C31B—Cl11—C31	36.2 (7)
C13—C14—H14	119.7	Cl11—C31—H31A	109.5
C14—C15—C16	119.2 (4)	Cl11—C31—H31B	109.5
C14—C15—H15	120.4	H31A—C31—H31B	108.1
C16—C15—H15	120.4	Cl11—C31B—H31C	108.4
N11—C16—C15	121.6 (4)	Cl11—C31B—H31D	108.4
N11—C16—H16	119.2	H31C—C31B—H31D	107.4
			
N21—Sn1—N11—C16	177.5 (3)	Cl4—Sn1—N21—C26	−92.4 (3)
Cl2—Sn1—N11—C16	−4.9 (3)	Cl3—Sn1—N21—C26	93.7 (3)
Cl1—Sn1—N11—C16	179.1 (3)	N11—Sn1—N21—C22	3.2 (2)
Cl4—Sn1—N11—C16	89.3 (3)	Cl2—Sn1—N21—C22	−8.7 (6)
Cl3—Sn1—N11—C16	−96.4 (3)	Cl1—Sn1—N21—C22	−176.4 (2)
N21—Sn1—N11—C12	−2.9 (2)	Cl4—Sn1—N21—C22	89.7 (2)
Cl2—Sn1—N11—C12	174.6 (2)	Cl3—Sn1—N21—C22	−84.2 (2)
Cl1—Sn1—N11—C12	−1.3 (5)	C26—N21—C22—C23	−0.7 (5)
Cl4—Sn1—N11—C12	−91.2 (2)	Sn1—N21—C22—C23	177.4 (3)
Cl3—Sn1—N11—C12	83.1 (2)	C26—N21—C22—C12	178.7 (3)
C16—N11—C12—C13	1.2 (5)	Sn1—N21—C22—C12	−3.2 (4)
Sn1—N11—C12—C13	−178.4 (3)	N11—C12—C22—N21	0.5 (5)
C16—N11—C12—C22	−178.1 (3)	C13—C12—C22—N21	−178.7 (3)
Sn1—N11—C12—C22	2.4 (4)	N11—C12—C22—C23	180.0 (3)
N11—C12—C13—C14	−1.4 (5)	C13—C12—C22—C23	0.7 (5)
C22—C12—C13—C14	177.9 (3)	N21—C22—C23—C24	0.7 (5)
N11—C12—C13—C17	178.8 (3)	C12—C22—C23—C24	−178.7 (3)
C22—C12—C13—C17	−2.0 (5)	N21—C22—C23—C27	−179.7 (3)
C12—C13—C14—C15	−0.1 (6)	C12—C22—C23—C27	1.0 (5)
C17—C13—C14—C15	179.7 (4)	C22—C23—C24—C25	−0.3 (6)
C13—C14—C15—C16	1.7 (6)	C27—C23—C24—C25	−179.9 (4)
C12—N11—C16—C15	0.5 (6)	C23—C24—C25—C26	−0.2 (6)
Sn1—N11—C16—C15	−180.0 (3)	C22—N21—C26—C25	0.1 (6)
C14—C15—C16—N11	−2.0 (7)	Sn1—N21—C26—C25	−177.7 (3)
C12—C13—C17—C27	1.7 (6)	C24—C25—C26—N21	0.3 (6)
C14—C13—C17—C27	−178.1 (4)	C13—C17—C27—C23	−0.1 (6)
N11—Sn1—N21—C26	−178.8 (3)	C22—C23—C27—C17	−1.3 (6)
Cl2—Sn1—N21—C26	169.2 (3)	C24—C23—C27—C17	178.3 (4)
Cl1—Sn1—N21—C26	1.5 (3)		

Symmetry codes: (i) −*x*+1/2, *y*−1/2, *z*; (ii) −*x*+1, −*y*, −*z*+1.
